# Preoperative management in octogenarian patients with rectal cancer

**DOI:** 10.1016/j.heliyon.2024.e41469

**Published:** 2024-12-25

**Authors:** Arthur M. Damasceno, Rubens Kesley, Marcus Valadão, Fabrício Braga, Cristiane A. D'Almeida, Marcos B. Pitombo

**Affiliations:** a Post-Graduate Program in Medical Sciences, Rio de Janeiro State University (UERJ), 444 Prof Manuel de Abreu Avenue, Rio de Janeiro, RJ, 20550-170, Brazil; b Abdominal Surgery Department and Nutritional and Dietetic Service, Brazil National Cancer Institute (INCA), 23 Praça da Cruz Vermelha, Rio de Janeiro, RJ, 20230-130, Brazil; c Laboratório de Performance Humana, 1 Largo do Ibam, Rio de Janeiro, RJ, 22271-070, Brazil

## Abstract

**Background:**

In recent years, the rise in average lifespan has been linked to an increase in the occurrence of diseases associated with aging worldwide. Rectal tumors often occur in elderly patients.

**Methods:**

Between January and August 2024, 6 experts in colorectal cancer met to develop an algorithm to organize the interdisciplinary and multimodal preoperative approaches in the octogenarian population with rectal cancer. To develop the algorithm, we conducted a straightforward search within the PubMed database and also reviewed the citations of the most pertinent articles we discovered. The quality of the methods used in the final selection of 76 sources was evaluated, every single source was scrutinized and analyzed, and a team of six experts created an algorithm.

**Results:**

An algorithm for preoperative management of octogenarian patients with rectal cancer was created to encapsulate essential information and provide a contemporary resource for physicians, surgeons, physiotherapists, and nutritionists to utilize in optimal clinical practice.

**Conclusions:**

Octogenarian patients with rectal cancer are special-character groups and require specific preoperative management to better the surgical outcome.

## 1 Background

Life expectancy has increased throughout the world over the last few years, bringing with it the incidence of age-related diseases. Most solid tumors occur more frequently in older age, including rectal tumors [1]. Among men, colorectal cancer is the third most frequently diagnosed cancer, representing 10 % of all cases. For women, it is the second most prevalent cancer, with an incidence rate of 9.2 % [2]. Generally, rectal cancer diagnosis is considered to do psychological damage and also arise discomfort symptoms. Along with other comorbidities, the functionality, and staging of the disease should also be taken into consideration in elderly patients who have been diagnosed with rectal cancer. The results of such an evaluation are also compared with the evaluation by the oncologist to arrive at the decision whether chemotherapy and radiotherapy are to be done only or before surgical resection as neo-adjuvant therapy.

Since in the vast majority of cases, even elderly patients older than 80, with rectal cancer are potentially curable with surgical resection, proper preoperative management of comorbidities, functionality, and nutrition may decrease the morbidity of these patients and allow most to survive and benefit following such surgical treatment. This paper reviews issues of preoperative management in the octogenarian patient with rectal cancer and makes suggestions as to how the operative risks in this group of patients might be lessened.

## 2 Methods

Between January 2023 and August 2024, 6 experts in colorectal cancer met to develop an algorithm to organize the interdisciplinary and multimodal preoperative approaches in the octogenarian population with rectal cancer.

For the development of this algorithm, PubMed database searched up to August 31, 2024. Search terms and their combinations included “rectum cancer”, “rectum tumor”, “anemia”, “cancer nutrition”, “multimodal treatment”, “surgical risk” and “octogenarians”. One hundred and fifty seven articles were found, 81 of which were removed because they were not relevant to the proposed topic or they had poor quality design or they did not provide practical approaches to the topic. Following the compilation of a final list comprising 76 sources, the quality of evidence from each source was evaluated and analyzed by a panel of six experts to aid in the creation of the algorithm.

To classify the strength of the evidence and the degree of recommendations, a modified version of the Grading System from the Infectious Diseases Society of America along with the United States Public Health Service was employed (Table 1) [3].

**Table 1:** Levels of evidence and grade of recommendation.

Levels of evidence	
I	Evidence from at least on large on large randomized controlled trial with good methodological quality (low potential bias) or meta-analyses of well-conducted randomized trials without sample heterogeneity
II	Small randomized trials or large randomized trials with suspected bias (poor methodological quality), meta-analyses of these trials, or trials with demonstrated sample heterogeneity
III	Prospective cohort studies
IV	Retrospective cohort or case-control studies
V	Studies without control groups, case reports, and expert advice
Grade of recommendation	
A	Strong evidence of efficacy with significant clinical benefit; strongly recommended
B	Strong of moderate evidence os efficacy but limited clinical benefit; usually recommended
C	Insufficient evidence of efficacy or benefit does not outweight risk or disadvantages (ie, adverse events, costs, another factors); recommended in some cases
D	Moderate evidence of ineffectiveness or occurrence of adverse outcomes; rarely recommended
E	Strong evidence of ineffectiveness or occurrence of adverse outcomes; never recommended

A novel algorithm has been created to extract essential information into a modern tool, assisting surgeons and doctors in providing optimal care to patients in their eighties with rectal cancer. All the topics were discussed in a video conference meeting for final consensus by all the 06 experts.

## 3 The first preoperative evaluation

### 3.1 Multimodal prehabilitation (relaxation, exercise and nutrition)

Octogenarian patients are complicated and should be assessed by a multidisciplinary team so as to adequately address psychological, physical, and nutritional dimensions.

This can include elective psychological assessment, which can hugely mitigate the risks to surgery. The depression and anxiety risks in such cases are very high [4], and hence monitoring by a psychologist, and at times by a psychiatrist, becomes very important toward improving their well-being and adhering to required perioperative care [5]. The relation of depression and anxiety with infection, wound healing, and length of stay is well established [[6], [7], [8]]. Psychological surveillance links to better postoperative outcomes [9].

In addition to psychological support, it is also essential to assess functionality because old age itself is not a contraindication for colorectal surgery [10]. Several research works point towards the better postoperative progress of those patients undergoing preoperative exercises [11,12]. According to Faithfull et al. [10], upon the review of several studies, it was evidenced that either physical or multimodal supportive therapy brings benefits over the approach where these are not available.

**Fig. 1:**
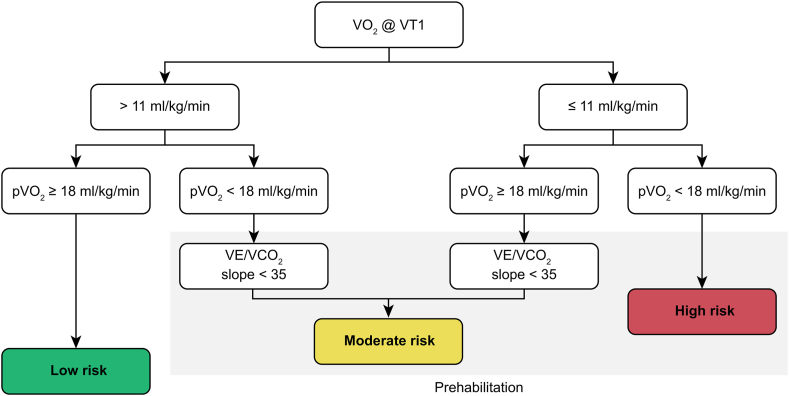
Risk stratification for gastrointestinal surgery.

The indication of a preoperative exercise program is based on the risk stratification obtained from the cardiopulmonary exercise test [13]. Three parameters of this test are used for risk classification: volume of oxygen uptake (VO_2_) at the first ventilatory threshold (marker of the beginning of the use of anaerobic energy sources); VO_2_ at peak effort; and the relationship between pulmonary ventilation and oxygen production (VE/VCO_2_ slope). Fig. 1 shows how, in our experience, we have integrated these three variables to classify the operative risk and choose the prehabilitation program.

Any such exercise program should contain aerobic, strength, neuromotor, and flexibility training. Individuals with documented respiratory muscle weakness benefit from the addition of respiratory muscle training.

#### 3.1.1 Respiratory muscle training

For training of the inspiratory muscles, it is mandatory to measure the maximal inspiratory pressure (Pimax). This measurement is performed noninvasively and rapidly. We suggest that the measurements be recorded with slow motion video for greater accuracy.

The patient is seated with the trunk erect (90° from the thighs) and arms relaxed on the side of the body while wearing a nose clip. The patient's lips are sealed around the tip of the tube connected to the manometer device. After a maximum expiration, the patient is instructed to perform a deep inspiration with the system occluded. Three valid measurements are obtained, and the highest absolute value is recorded (with the negative sign). The value is expressed in cmH_2_O.

The improvement in the respiratory muscles via training the inspiratory muscles is directed toward a reduction of pulmonary complications after major surgery on the thoracic or upper abdominal cavities. It has been proved that inspiratory muscle training reduces atelectasis, pneumonia, and the number of days a patient is forced to stay in the hospital. The regimen is shown in Table 2 as described by Braga [14] and adapted from Dronkers [15].

**Table 2:** Respiratory muscle training program.

Minimal recommended training length	Weekly rate	Daily rate	Intensity (Power breath®)
2 weeks	5–7x	2× of 5–30min at least 15 breathing cycles for training	Start at 40 % of MIP^a^. Add 2cmH_2_O for the next session every time Borg <5/10 at the end of the training

a MIP: Maximal inspiratory pressure.

#### 3.1.2 Nutrition

Good nutritional monitoring improves the benefits of this preoperative preparation [16,17]. Given weight loss, reduction in muscle mass, and dietary shifts, which also tend to increase with age, patients with CRC might feature poor nutritional statuses. Preoperative risk factors afflict this elderly subset of the CRC surgery patient collective, where most members are far more vulnerable than their younger counterparts to a number of preoperative risks that do not feature universal applicability in younger patients [18].

Nutrition status is the most important predictor to assess the risk of complications after the surgical procedure has been done. Nutrition's contribution to treatment modalities for CRC has been extensively researched, with multimodal interventions during the treatment period constituting an extended arm for the early preparation of patients to adequately face the short and long-term effects of surgery [[19], [20], [21]].

At the time of diagnosis, malnutrition affects between 15 and 40 % of cancer patients, and between 80 and 90 % of patients at a more advanced stage [22]. Within a patient grouping with CRC, this percentage will vary from 45 to 60 % [23]. Malnutrition has negative effects that induce a decrease in both the tolerance and efficacy of treatment in cancer patients [24]. It increases the likelihood of clinical and instrumental complications in surgery [25]. It lengthens stays in the hospital, which correlate with increasing health cost expenditure [26]. According to some reports, malnutrition is the cause of death for 10 %–20 % of individuals with cancer, rather than the disease itself [27].

Screening recommendations for elderly cancer patients are not well defined. However, following a cancer diagnosis in an old patient, the evaluation will be carefully conducted on how the comorbidity, physical function, and psycho-social health might influence the course of treatment. According to ESPEN, the nutritional intake should be assessed at an early stage of the disorder. Thus, nutrition intake, weight change and BMI have been recommended for regular evaluation starting from cancer diagnosis repeating their frequency depending up on the stability of clinical situation [28]. The advice for patients with a positive screening test is an objective quantitative appraisal of the nutrition intake, symptoms of nutrition impact, muscle mass, a physical performance as well as systemic inflammation level [28].

A group of specialists in geriatric oncology suggested using the Comprehensive Geriatric Assessment to determine which elderly cancer patients might gain from undergoing treatment and which might find palliative care to be a more suitable option [[29], [30], [31]]. This assessment identifies changeable and reversible conditions, forecasts the potential for treatment-related side effects, and evaluates the available family support against what is necessary for implementing the treatment plan. Regrettably, there is a scarcity of formal studies examining the use of the Comprehensive Geriatric Assessment alongside treatment protocols. More research is needed to explore how effectively the CGA can predict and guide treatment decisions for older cancer patients [31].

The quality of life and daily activities of a patient can be greatly affected by the symptoms of cancer and the side effects of its therapies. Because elderly patients often have several declines in physiological systems due to age, this may be related to a decline in physical resilience and a heightened vulnerability to health deterioration when faced with factors that cause stress such as cancer and its associated therapies. Therefore, it has to be considered that older adults with cancer need to contact their healthcare providers more frequently for reporting symptoms and they must receive clear guidance determining the timing and method to request help from their healthcare specialists [32].

Postoperative complications can develop in as many as half of CRC patients, leading to increased illness and death rates, reduced quality of life related to health, and elevated healthcare expenses. The severity and number of complications are strongly associated to the individual's functional ability before surgery, their nutritional health, mental state, and cigarette use condition [33]. In addition to malnutrition, we should also notice obesity because it results in worse outcomes in the patients with gastrointestinal neoplasms [34] and it poses a risk for heart and kidney disorders, diabetes, and also postoperative mobility impairment.

Patients scheduled for elective surgeries should undergo routine screening and preoperative nutritional assessment to categorize their risk and nutritional status. Older patients are frequently more fragile and require personalized preoperative management that can prevent adverse complications and improving clinical outcomes [35].

The nutritional management in cancer patients scheduled for surgery involved assessing their nutritional status before, during, and after the operation and formulations which involved the evaluation of nutritional status, nutritional counseling, maximizing protein, and caloric intake, starting nutritional support promptly with oral supplements, as well as enteral or parenteral feeding, utilizing the most effective dietary treatment for the patient's current medical condition [27,36].

For individuals with cancer, when Total Energy Expenditure (TEE) is not specifically measured, it is generally assumed to be similar to that of people without cancer, usually ranging from 25 to 30 kilocalories per kilogram of body weight each day. Daily protein intake should be more than 1 g per kilogram of body weight, aiming for up to 1.5 g per kilogram per day if possible [28].

Immunonutrition is a well-known intervention for elective surgery, and in oncology [37,38] it reduces hospitalization time and the rate of complications, especially infections [39,40]. The supplementation of immunomodulatory agents within the perioperative setting is critical for the nourished and malnourished patient. In well-nourished patients, taking preoperative nutritional supplements enriched with omega-3 fatty acids can potentially affect the immune system and lessen the body's inflammatory reaction following surgery, thereby impacting the metabolic adjustments associated with elective surgical procedures. Preoperative intervention benefits the malnourished not only with metabolic but more robust immune effects and a lower risk of infection after major surgery [38].

Analyzing the therapeutic impact of immunonutrition is challenging due to the difficulty in identifying which specific nutrients, if any, offer benefits. Eicosapentaenoic acid (EPA) and docosahexaenoic acid (DHA), which are very long-chain n-3 fatty acids, are easily absorbed into body tissues. This absorption results in a reduction in the synthesis of substances that promote inflammation, by interfering with arachidonic acid metabolism [41].

EPA is a type of long-chain polyunsaturated fatty acid that belongs to the omega-3 group. It is known for its ability to promote anabolism and modulate the immune system, making it beneficial during the recovery period after surgery. Adding omega-3 to one's diet has demonstrated advantages for patients who are critically ill with acute respiratory distress syndrome as well as for those undergoing significant abdominal surgeries [[42], [43], [44], [45]]. In a double-blind randomized study, enteral nutrition containing EPA appeared to help maintain body weight and lean body mass following oesophagectomy [46,47], proposing that EPA offers advantages for individuals undergoing significant surgical procedures.

The immunonutrition protocol should be started 5–7 days before surgery (500–1000 mL/day) [27,48]. Therefore, early recognition of nutritional status is decisive for improving the response to treatment, with better functional capacity, higher survival rate, fewer postoperative complications, and greater likelihood of an overall positive surgical outcome.

We still do not have in the literature the appropriate frequency for monitoring older cancer patients, although we know that regular and close nutritional monitoring can alleviate symptoms, maintain nutritional status and consequently tolerance to treatment and quality of life.

These three interconnected therapies (psychology, exercise, and nutrition) take into account that age alone is not a determinant for an unfavorable surgical outcome [10]. They can exponentially increase functionality, improving the likelihood of a positive surgical outcome.

### 3.2 Anemia management

The World Health Organization characterizes anemia as having hemoglobin levels below 12 g/dL in non-pregnant women and under 13 g/dL in men [49]. Anemia prevalence in CRC patients varies between 30 % and 75 % [50], with 25 % of CRC patients being diagnosed with moderate-severe anemia (Hb < 10 g/dL) [51].

Although anemia is a common sign in the diagnostic period of CRC, its preoperative management is key to a better surgical approach, since patients who are transfused are admitted for longer times, are at greater risk of infection and lung injury, stay longer in intensive care unit [[52], [53], [54]], and also have more recurrence of cancer [55,56].

In regard to anemia etiology, CRC patients most commonly suffer anemia resulting from a lack of iron and anemia related to inflammation [57,58] (in which immune activation and suppression of erythropoiesis coexist) plus both of them. Less frequent causative factors include vitamin B12 deficiency, chronic kidney disease, and other hematologic reasons. Taking appropriate measures to treat anemia is very important to reduce the perioperative morbidity of the patient [59,60].

The treatment of anemia caused by a lack of iron involves replacing it with iron supplements. In mild cases and those with a late surgical schedule, oral iron replacement can be evaluated, taking into account that its absorption may be affected by feeding, inflammation, and medications. In addition, adherence to oral iron replacement is less than 50 % [61] due to gastrointestinal complications, and the response is poor when there is active bleeding. Venous iron is absorbed more in an effective and more rapid manner than oral iron; therefore, intravenous (IV) administration is the best means of treatment when the oral route is contraindicated/inadequate, or in the case of marked anemia (Hb < 10 g/dL). The occurrence of negative reactions is less frequent with intravenous iron infusions compared to allogeneic blood transfusion [62]. The risk-benefit ratio of iron replacement always favors reducing the risk of blood transfusion and its complications. There are various formulations and regimens for IV iron infusion, as reviewed by Warner et al. [63].

Another potentially helpful treatment is human erythropoietin (erythropoiesis-stimulating agents - ESAs), which accelerates recovery from anemia when combined with IV iron. In addition to iron deficiency, anemia can also be caused by inflammation or chronic diseases and is also related to immune activation, inadequacy of erythropoiesis, and very high levels of hepcidin. Once a diagnosis of anemia of inflammation has been made, then consideration should be given to ESAs after IV iron replacement. It significantly raises the levels of hemoglobin in patients subjected to gastrointestinal surgery or those planned for CRC surgery and, hence, reduces transfusion requirements [[64], [65], [66], [67], [68]]. When given after IV iron replacement for anemia of inflammation, it may be administered in a dosage of 600 U/kg subcutaneously thrice weekly [63].

### 3.3 Control of comorbidities and symptoms

In the preoperative preparation, besides controlling the patient's disease, it is also key to manage heart-related conditions, diabetes, long-term kidney issues, and tobacco use (smoking cessation) to optimize the success of an operation and later improve its quality of life [69,70]. It is also important to control symptoms such as anemia (already mentioned) and pain. Pain is present in 70 % of patients with CRC [71]. Controlling underlying diseases and symptoms, which often requires joint monitoring with other specialists, increases quality of life, adherence to treatment and surgical success.

## 4 Presurgical return after therapeutic optimization

After the evaluation period and adequate treatment of comorbidities and symptoms, some symptoms, such as pain and bleeding, may remain uncorrected but may improve with tumor removal. Perioperative blood transfusion may be needed especially for those patients with heart and lung disease states with far less tolerance to anemia. Surgical outcomes are better when rectal cancer is approached by a specialist surgeon [72]. Valadão et al. [73] recently published a review on surgical approaches for the middle and lower rectum and described in detail the surgical technique to be adopted. There are several surgical risk scores for colorectal neoplasms that can aid decision-making by the multidisciplinary team, in conjunction with the family, about which approach proposed by an experienced surgeon to adopt.

### 4.1 Surgical risk assessment

Preoperative evaluation requires great care, especially for more fragile patients. Surgical treatment of rectal cancer tends to be more complex and have greater morbidity than that of colon cancer, even though both cancers are usually grouped as CRC.

Beginning with the first preoperative visit, several interdisciplinary approaches are needed to achieve the best possible treatment and clinical strategies to reduce the risks of surgical approaches.

Once the patient is thoroughly evaluated, their surgical risk is analyzed. The results of this analysis will inform both the choice of surgical technique and the dialog with the patient to collaboratively define actions.

Several methods of surgical risk stratification are available that can improve the prediction of possible complications related to the surgical procedure, including mortality. The most commonly used are SRS [74], AFC score [75], P-POSSUM) [76], Revised ACPGBI Score [77], and Surgical Risk Calculator (ACS-NSQIP) [78,79], some of which are specifically aimed at CRC [68,70,71].

SRS [67] evaluates information from the CEPOD grade (1–4), ASA grade (1–5), and BUPA operative grade (1–5) and analyzes the urgency of the surgery from elective to emergency, the size of the surgery from small to large, and the functionality from best to worst. Its score ranges from 3 to 14 points, higher scores indicating higher surgical mortality. This surgical risk takes into account only preoperative factors with mortality outcomes.

Another surgical risk stratification method is the AFC score [75], which evaluates four risk factors: age over 70 years, neurologic comorbidity, malnutrition (evaluated by the loss of weight greater than 10 % within 6 months), and emergency surgery. In this score, the likelihood of death is 0.5–2% in its absence or in one of the risk factors, 10 % when there are two risk factors, 20 % when there are three risk factors, and 50 % when there are four risk factors. This surgical risk assessment method is easily performed using preoperative factors and is exclusive to colorectal resection for cancer and diverticulitis.

P-POSSUM [76] evaluates several parameters separated into two scores. The first is the physiological score, which evaluates age, cardiac and respiratory manifestations, Electrocadiogram findings, SBP, HR, Hb, WBC, URE, Na, K, and GCS. The second is the operative severity score, which evaluates procedures number, surgical blood loss, peritoneal contamination, malignant status, and CEPOD grade. This surgical risk stratification method evaluates preoperative and intraoperative parameters but is not simple to use.

The new ACPGBI Score [77] developed in 2010 evaluates five parameters, age, tumor stage (Dukes’ staging system), urgency of surgery (elective/urgent/emergency), and surgical procedure. This method is also specific to colorectal procedures and considers preoperative factors.

The American College of Surgeons developed an assessment tool, Surgical Risk Calculator [78,79], that evaluates several risk factors by procedure: age, sex, functional status, emergency surgery (yes or no), chronic use of steroids for chronic conditions, ascites within 30 days of the procedure, systemic sepsis within 48 h post-op, dependence on mechanical ventilation, metastatic cancer, diabetes, hypertension, CHF up to 30 days post-surgery, dyspnea, smoking within the last year, severe COPD, with or without dialysis, acute renal failure, and BMI. Of the most commonly used surgical risk stratification methods, it is the only one that assesses the chances of several complications, it is the only one that considers likelihoods for several complications - hemodialysis, pneumonia, and urinary infection - with mortality. In addition, if the surgeon believes that the result of the evaluation is an underestimate of risk, the assessed risk can be increased according to the technical difficulty of the case.

P-POSSUM is an important surgical risk stratification method and a milestone within surgical oncology, but it is impractical because many of the variables are intraoperative and complex to adopt. There are data showing that P-POSSUM tends to overestimate the likelihood of illness and death in young individuals undergoing planned colorectal operations [76]. The new ACPGBI score has shown to be easier and more precise at predicting surgical mortality in colorectal cancer surgeries compared to P-POSSUM [77]. SRS is equivalent to P-POSSUM for the evaluation of high-risk surgeries but is easier to use because it encompasses fewer variables, and only preoperative ones. Neither SRS nor P-POSSUM are exclusively intended for the evaluation of colorectal surgeries.

Like the new ACPGBI score, the AFC score evaluates only colorectal surgeries. Compared with P-POSSUM, the AFC score provides similar predictions of operative mortality but is much simpler to perform.

There are no comparisons of the Surgical Risk Calculator with established scores in the literature. This score considers many preoperative variables. Increasing the standard deviation for the procedure to be performed allows the surgeon to include the surgical difficulty and to predict the likelihood of perioperative complications such as pneumonia, urinary tract infection, and mortality.

If surgical risk analysis shows that a patient older than 80 years is eligible for elective rectal cancer surgery, he or she may undergo an additional evaluation developed by Damasceno et al. [80] that considers three risk factors CEA greater than 5 g/dL and ALB less than 3.5 g/dL, and reoperation. Each factor is assigned 1 point, so the possible final scores are 0, 1, 2, and 3, which have had 30-day survival rates of 97.2 %, 90.5 %, 70.5 %, and 29.3 %, respectively. Using Damasceno's score [80] for patients older than 80 years provides more data and risks that are complementary to the previous evaluation. This score was developed precisely to assess which healthier elderly patients are likelier to die from surgery and which have favorable surgical risks. This score provides a better basis for therapeutic decisions by the team, in addition to serving as a prognostic tool in this specific scenario (octogenarian patients with rectal cancer). Table 3 compares the different surgical risks in octogenarians, and the variables are described in Table 4.

**Table 3:** Comparison between surgical risks in rectal cancer surgery in octagenarians.

Surgical risk	Types of surgeries	Variables	Form of use	What is evaluated
SRS [74]	All types of surgery	- CEPOD grade^a^ - ASA grade^a^ - BUPA operative grade^a^	Preoperative score	Postoperative mortality
AFC Score [75]	Colorectal resection in diverticulitis and cancer	- Age >70 years - Neurologic comorbidity - Loss of weight >10 % in <6 months - Emergency surgery	Preoperative score	Postoperative mortality
P-POSSUM [76]	Cancer surgery in general	- Age - Cardiac manifestations - Respiratory manifestations - Electrocadiogram findings^a^ - SBP - HR - Hb - WBC - URE - Na - K - GCS - No. procedures - Surgical blood loss∗ - Peritoneal contamination - Malignant status - CEPOD grade	Perioperative score	Postoperative mortality and surgical complications
New ACPGBI Score [77]	Colorectal surgeries	- Age - ASA Status - Dukes stage^a^ - Operative urgency^a^ - Operative procedure^a^	Preoperative score	Postoperative mortality
ACS-NSQIP [78,79]	All types of surgery	- Age - Sex - Functional status - Emergency surgery - ASA class - Chronic use of steroids for chronic conditions - Ascites within 30 days prior of the procedure - Systemic sepsis within 48 h post-op - Dependence on mechanical ventilation - Metastatic cancer - Diabetes - Hypertension requiring medication - CHF up to 30 days post-surgery - Dyspnea - Smoking within the last year - Severe COPD - With or without dialysis - Acute renal failure - BMI	Preoperative score	Postoperative mortality and surgical complications
Damasceno's score [80]	Surgery for rectal cancer in octogenarians	- CEA > 5 g/dL - Albumin <3,5 g/dL - Reoperation	Perioperative score after initial assessment with other surgical risks	Postoperative mortality

Table comparing different surgical risks and their variables in the approach to rectal cancer.

a Variables described in Table 4.

**Table 4:** Description of the variables marked in Table 3.

	Description
CEPOD grade	
Elective	Routine booked non-urgent, e.g. varicose veins or hernia
Scheduled	Booked admission, e.g. cancer of the colon or AAA
Urgent	Cases requiring treatment within 24–48 h of admission, e.g. obstructed colon
Emergency	Cases requiring immediate treatment, e.g. ruptured AAA
ASA	
I	No systemic disease
II	Mild systemic disease
III	Systemic disease affecting activity
IV	Serious disease but not moribund
V	Moribund, not expected to survive
BUPA operative grade	
Minor	Removal of sebaceous cyst, skin lesions, oesophagogastric duodenoscopy
Intermediate	Unilateral varicose veins, unilateral hernia repair, colonoscopy
Major	Appendicectomy, open cholecystectomy
Major plus	Gastrectomy, any colectomy, laparoscopic cholecystectomy
Complex major	Carotid endarterectomy, AAA repair, limb salvage, anterior resection, oesophagectomy
Electrocardiogram findings	
	Normal
	Atrial fibrillation (rate 60–90)
	Any other abnormal rhythm or ≥ 5 ectopics/min Q saver or ST/T wave changes
Surgical blood loss	
	≤100 mL
	101–500 mL
	501–999 mL
	≥1000 mL
Dukes	
A	The cancer is in the inner lining of the bowel. Or it is slightly growing into the muscle layer.
B	The cancer has grown through the muscle layer of the bowel.
C	The cancer has spread to at least 1 lymph node close to the bowel.
D	The cancer has spread to another part of the body, such as the liver, lungs or bones.
Operative Urgency	
	Elective
	Urgent
	Emergency
Operative procedure	
	Right hemicolectomy
	Transverse colectomy
	Left hemicolectomy
	Sigmoid colectomy
	Subtotal/Total colectomy
	Anterior resection
	Abdominoperineal resection (APER)
	Hartmann's procedure
	Palliative stoma
	Examination under anaesthetic (EUA)/Laparotomy/Laparoscopy only

**Fig. 2:**
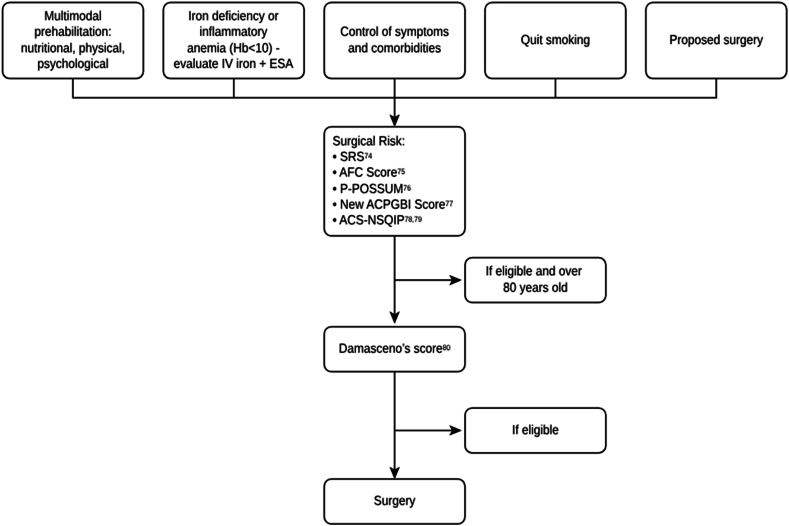
Approach to octagenarians with rectal cancer.

Due to the complexity of the treatment of multiple comorbidities and of the different approaches that can improve the surgical outcome, combined with the surgical risk, we developed an algorithm to organize the interdisciplinary and multimodal approaches in the octogenarian population with rectal cancer (Fig. 2). Table 5 demonstrates recommendation grade and evidence level of suggested algorithm.

**Table 5:** Levels of evidence and grade of recommendation of the suggested algorithm.

Interdisciplinary and multimodal approaches	Level of evidence	Grade of recommendation
Multimodal prehabilitation	III	B
Iron deficiency of inflammatory anemia	II	B
Control of symptoms and comorbidities	III	B
Quit smoking	III	B
Proposed surgery	V	B
Surgical Risk	I	A
Damasceno's score	IV	B

## 5 Discussion

When pre-surgical optimization measures were evaluated separately for rectal cancer, unequivocal clinical benefit was shown. On the other hand, incorporations of those aforementioned pre-surgical optimization measures with traditional preoperative optimization tools have never been evaluated, especially for patients older than 80 years. Therefore, the proposal of this review to add pre-surgical optimization measures to traditional pre-operative risk assessment tools and Damasceno's score can make this pre-operative assessment more accurate for the doctor's decision-making. It is important to state that the Damasceno's score represents an instrument in this type of population (octogenarians with rectal cancer undergoing traditional surgical risk), which should be used in a complementary way to traditional scores, aiming to increase the efficiency of preoperative assessment.

Furthermore, it should be pointed out that this profile of patients (octogenarians with rectal cancer) are usually excluded from clinical studies, which restricts data on this topic, making it difficult to critically analyze data from the literature. It is worth highlighting that the present study refers to a theoretical algorithm proposal based on literature (through evidence of the use of different assessment tools in isolation), with no studies published to date with the objective of validating this flowchart.

There are few studies available for surgical approach in the population over 80 years of age with rectal cancer, limiting this analysis. In addition, this evaluation is limited only to seniors over 80 years of age. It is worth noting that no study has been carried out for this preoperative preparation in this population, which values the construction of this algorithm.

That said, new trials will be needed that encompass this specific group of patients (octogenarians with rectal cancer with traditional surgical risk excluded), using the algorithm proposed in the present study in order to validate this recommendation.

## 6 Conclusion

A combination of adequate preoperative preparation, perioperative risk assessment and decision-making with the patient consult leads to a lower rate of complications and higher success rate of surgery. These aforementioned measures would be helpful in optimizing surgical outcomes in octogenarian's rectal cancer patients.

## CRediT authorship contribution statement

**Arthur M. Damasceno:** Writing – original draft, Methodology, Data curation, Conceptualization. **Rubens Kesley:** Writing – review & editing, Supervision. **Marcus Valadão:** Writing – review & editing, Visualization. **Fabrício Braga:** Writing – original draft. **Cristiane A. D'Almeida:** Writing – original draft. **Marcos B. Pitombo:** Writing – review & editing, Supervision.

## Informed consent

Informant consent form not applicable to this review article.

## Ethics approval

Approval was obtained from the Institutional Review Board of the National Cancer Institute of Brazil and the Rio de Janeiro State University, Local Committee of Ethics, respectively. The research adhered to the principles outlined in the Declaration of Helsinki.

## Data availability statement

This article does not involve data sharing because the study did not generate or examine any new data.

## Lay summary

Performing surgery on patients in their eighties who have rectal cancer constitutes one of the most difficult challenges for the surgeon and interdisciplinary team; however, proper preoperative management can substantially lower the incidence of complications and surgical mortality among these patients. The current review aims to develop a practical guide for preparing elderly patients with rectal cancer before surgery to ensure optimal surgical outcomes.

## Precis

An algorithm was developed to organize the interdisciplinary and multimodal approaches to rectal cancer in the octogenarian population.

## Declaration of competing interest

The authors declare that they have no known competing financial interests or personal relationships that could have appeared to influence the work reported in this paper.
